# Duration of dam contact had a long effect on calf rumen microbiota without affecting growth

**DOI:** 10.3389/fvets.2025.1548892

**Published:** 2025-05-12

**Authors:** Laurianne Voland, Abimael Ortiz-Chura, Jeremy Tournayre, Bruno Martin, Matthieu Bouchon, Alessandra Nicolao, Dominique Pomiès, Diego P. Morgavi, Milka Popova

**Affiliations:** ^1^Université Clermont Auvergne, INRAE, VetAgro Sup, UMR Herbivores, Saint-Genès-Champanelle, France; ^2^INRAE, UE Herbipôle, Saint-Genès-Champanelle, France; ^3^DAFNAE (Dipartimento di Agronomia, Animali, Alimenti, Risorse naturali e Ambiente), University of Padova, Legnaro, Italy

**Keywords:** cow-calf contact, rumen microbiota, pathogens, dysbiosis, early-life

## Abstract

**Introduction:**

Separating calves from their mothers at birth is linked to calf welfare issues and disturbances in the mother-calf relationship. It can also disrupt the maturation of the digestive tract, affecting calf health. It has been demonstrated that separation at weaning allows for the optimal establishment of the ruminal microbiota, whereas separation at birth alters colonization dynamics. We postulated that 4 weeks of cow-calf contact, a potentially more socially acceptable, and economically pragmatic, management practice, would induce a similar development of ruminal microbiota to that observed with separation at weaning, thereby conferring benefits on calf health and growth.

**Methods:**

We studied three groups of 14 cow-calf pairs (Holstein and Montbéliarde breeds) with different cow-calf separation times: 4 weeks of contact with the mother (Mixed group), immediate separation (at birth, Control group) and delayed separation at weaning (11 weeks, Dam group). Rumen microbial colonization was monitored in 9 calves per group at 3, 10, 13, and 20 weeks of age using a metataxonomic approach. Body weight, diarrhea and respiratory disease were recorded to assess the calves' overall health. Serum IgG concentrations were also monitored.

**Results:**

No differences were observed between the groups in diarrhea or IgG concentration. The incidence of respiratory disease was lower in calves that remained in contact with their dams until weaning. After separation, the Mixed group exhibited an increased average daily gain. The metataxonomic analysis demonstrated that as calves aged, there was an increase in richness, accompanied by a corresponding increase in the number of shared microbial species over time between all groups. Nevertheless, three discrete development pathways were identified in the rumen bacterial communities, as evidenced by the differences in beta diversity between the groups over time. Additionally, the presence of the mother had a favorable effect on the transfer of beneficial microbiota during the early stages of life. However, this was offset by the elevated detection of potential pathogens at a later age in the Dam group.

**Conclusion:**

In this study, the rearing method exerted a profound and enduring influence on the gastrointestinal microbiota, with no discernible negative impact on health.

## 1 Introduction

The separation of the calf from its mother soon after birth is the standard management practice method for the rearing of dairy calves on European dairy farms (1). The rationale for early separation encompasses enhanced milking efficiency, augmented milk sales, reduced labor and economic savings (2). Limiting the cow-calf bond through early separation has been shown to reduce stress at weaning and facilitate socialization of the calf with the farmer (3). Nevertheless, the potential downside of early separation is that it could lead to an increased prevalence of early-life digestive disorders and have an adverse impact on calf growth (1, 4). The practice of early separation is becoming increasingly controversial due to social pressures that emphasize the importance of the mother-calf relationship (3).

Alternative rearing systems, designated as Cow-Calf Contact (CCC) systems, are emerging as a potential means of enhancing animal welfare and harmonizing economic and social considerations (5). The CCC system allows for contact between cows and their calves for a portion of the day, which facilitates the bonding process between the dam and her calf while simultaneously ensuring optimal growth prior to and following weaning (5). Moreover, the contact with adult animals plays a significant role in the acquisition of gut microbes during the early stages of life (6–8). Acquisition of gut microbiota is observed to be modulated by maternal contact, the presence of conspecifics, as well as the farm environment, which includes milk and feed sources (6, 8, 9). However, the rumen microbiota of the dams was identified as the predominant (compared with other sites) source of bacteria (7.9%) and archaea (49.7%) in the calves' rumens (8). It has been proposed that prolonged contact with the dam facilitates a more gradual transition in microbial diversity within the rumen of dairy calves (10). In addition, greater diversity and maturity of rumen microbiota were observed in lambs (11) and goat kids (12) that had been reared with their mothers or in the presence of an adult companion, in comparison with their counterparts reared without an adult contact. Furthermore, the rapid acquisition of a diversified microbiota has been associated with beneficial health and performance implications later in life (13–16). It is evident that symbiotic gastrointestinal microbes play a pivotal role in maintaining animal health. They not only facilitate the breakdown of feed, but also engage in competitive interactions with pathogens and stimulate the development of the immune system, including the epithelial barrier and the mucosal immune system (17). It has been demonstrated that microbes that are essential for rumen functionality arrive at an early stage, even prior to the introduction of solid feed (18–20). By the age of 4 weeks, the major functional groups are already established (21).

The hypothesis that a reduction in the duration of the CCC period, as opposed to contact until weaning, would allow the acquisition of a diverse microbiota and, in turn, improve calf health and welfare with minimal negative effect on milk sales is a reasonable one to put forward. As an intermediate between immediate (at birth) and delayed (at weaning) separation, it is posited that a 4-week period of cow-calf contact would promote the development of a more diverse rumen microbiota, which would benefit calf health and growth to the same extent as contact until weaning with the dam. The objective of this study was to monitor the progression of rumen microbial colonization in these three management systems and to correlate it with growth and health parameters prior to and following weaning, up to 5 months of age.

## 2 Materials and methods

The study was conducted at the Herbipôle experimental farm (https://doi.org/10.15454/1.5572318050509348E12) of the French National Institute for Agriculture, Food and the Environment (INRAE), located in Marcenat, France (45.30°N, 2.84°E, 1,080 m a.s.l.), from 12 February to 1 August 2019.

### 2.1 Experimental design

The present study was conducted with 42 lactating dairy cows and their corresponding calves (21 Holstein and 21 Montbeliarde), recruited at parturition between 12 February and 5 May 2019. The dam-calf pairs were randomly assigned to one of three experimental groups by ensuring that there were no significant differences between the groups in terms of cow breed, lactation rank, date of calving and milk production index. During the period of parturition, the three groups of 14 cows each were housed in the same free-stall barn, which was divided into three equivalent pens. The cows had *ad libitum* access to a mixed ration (comprising 59% first-cut hay, 32% second-cut hay, and 9% concentrate) to which 2 kg of concentrate was added on an individual basis. From 5 May until the end of the trial, the cows grazed a permanent grass pasture and were supplemented with 2 kg/day of concentrate. All cows were milked in a 2 × 14 herringbone milking parlor (Delaval, France) at 06:30 and 15:30.

The three experimental groups were as follows: control group calves were separated from their dams within 6 h of birth; the Dam group calves were reared with their dams until weaning; and the Mixed group calves remained with their dams until 4 weeks of age, after which they were reared as in the Control group. Prior to the first sampling at 3 weeks (see below), five male calves were removed from each group for management reasons. The final number of nine calves per group consisted of all females and some males (Supplementary Table 1).

The Control group received 2 L of high-quality colostrum (Brix score > 24% measured by refractometry) immediately following birth. The calves were housed in individual pens for a period of 7 days, after which they were transferred to a collective straw-bedded pen with a hay rack. They were then provided with bulk milk and concentrate by automatic feeders in accordance with a pre-established feeding plan, which continued until the point of weaning (Supplementary Table 2). In the Dam group, each calf received colostrum from its dam, and the dam-calf pair was housed in individual pens for a period of 5 days. Subsequently, the calves were relocated to a collective straw-bedded pen with access to the dam's pen. Calves were permitted free access to the dams' pen during the daytime hours (09:00 to 15:30), and had therefore free access to pasture from 5 May but also to hay and concentrate in their pen, when access to dams was restricted (Supplementary Table 2). Following the removal of the males at 3 weeks of age, all cows remained in the pen, thereby the remaining nine calves had potential access to the 14 cows for suckling.

For the Mixed group, calves were reared as calves of the Dam group until 4.0 ± 0.5 weeks of age, except that they did not have access to pasture. After separation, mixed calves were moved to a separate group pen and fed bulk milk from an automatic milk dispenser as in the control group (Supplementary Table 2). Calves were abruptly weaned when they weighed ≥ 100 kg (on average at around 11 weeks of age) and moved to group pens. Weaning (in the 3 groups) and late separation (Mixed group) took place in batches. Every 2 weeks, calves were separated before their dam returned from Tuesday morning milking.

At weaning, the calves were moved to new pens, one for each group, to avoid mixing. In these pens, the calves were fed in groups the equivalent of 0.5 kg/day/calf of hay distributed in the evening with no refusal in the morning and 2.0 kg/day/calf of concentrate (Startivo, Centraliment, 15,006 Aurillac) distributed twice a day (Supplementary Table 2). Calves were fed this diet until the start of the grazing season. The calves were allowed to graze at an average age of 15 weeks, with the exception of the Dam group, who were allowed access to the pasture when they were still with the cows and calves were 4.6 ± 3.9 weeks of age. The characteristics and composition of the grazing plots have been reported previously (22) and are summarized in Supplementary Table 3.

### 2.2 Calf health assessment and sampling

#### 2.2.1 Weight and average daily gain

The calves were distributed across three experimental groups, to balance body weights within each group. Mean birth weight was 43.0 ± 5.50 kg for Control group, 42.4 ± 4.45 kg for Dam group and 38.8 ± 3.01 kg for Mixed group (mean ± SD). There were no significant differences between groups. Calves were weighed at birth and then weekly until 14 weeks of age. Average Daily Gain (ADG) was calculated at 3, 10, and 14 weeks following the equation:


$$\begin{array}{l} ADG\;i=\frac{\text{Weight week i }-\text{ Birth Weight }}{\text{ Date week i }-\text{ Birth Date }}\;\left(\frac{kg}{day}\right) \end{array}$$

where i = 3, 10, and 14 weeks.

#### 2.2.2 Respiratory and diarrhea disorders

Calves were assessed daily for health disorders by trained animal keepers. The occurrence of respiratory disease (runny nose, cough, dyspnoea) and diarrhea in calves was scored using a binary notation: zero for absence of disease and one for presence of disease.

For each type of disease, a prevalence frequency, up to 14 weeks of age, was calculated as follows:


$$\begin{array}{l} Prevalence=\\ \frac{\text{Cumulative number of individual recordings having a disorder during }14\text{ weeks}}{\text{n }=\text{ Total number of individual recordings in the population during }14\text{ weeks }}\times 100 \end{array}$$

where *n* = 112 for Control, *n* = 126 for Dam and *n* = 125 for Mixed groups.

The treatment of sick animals was conducted in accordance with a standard operating procedure that was in compliance with ethical guidelines and under the supervision of a veterinarian. The specific condition and treatments used were recorded in a health logbook. Only two animals were treated for respiratory disease with antibiotics: one from the Mixed group at week 6 for 5 days, and one from the Control group at week 6 for 3 days. One calf from the Control group was treated for diarrhea at week 3 for 1 day.

#### 2.2.3 Serum sampling

Blood samples were collected from all calves via jugular vein puncture into 10 mL Vacutainer tubes (Dutsher, France) at 48 h after birth, at week 3, prior to the separation of calves in the Mixed group, and finally at 10 weeks of age (1 week before weaning). Blood samples were immediately centrifuged at 3,000 × g for 20 min at 4°C and ~1 mL serum was aliquoted into 3 × 1.5 mL Eppendorf tubes, and stored at −20°C until IgG analysis. Serum samples were analyzed in the VetAgro Sup laboratory (Marcy l'Etoile, France), using a radial immunodiffusion method (Bovine IgG 1 Test from IDBiotech, Issoire, France) to determine the immunoglobulin G concentration (IgG, in mg/dL).

#### 2.2.4 Rumen sampling

Rumen fluid was collected from each calf at 3, 10, 13, and 20 weeks of age using an esophageal tube. The rationale behind starting the sampling at 3 weeks of age was to enable the major microbial groups to establish, which are variable from animal to animal early in life, thus facilitating the detection of any variation between treatments. The first few milliliters were discarded to avoid possible contamination by saliva. Samples (~50 mL) were filtered through a polyester monofilament fabric (250 μm mesh) and the filtrate was processed for microbial DNA extraction, volatile fatty acid (VFA) analysis and protozoan enumeration. Due to the constraints of the sampling methodology, it was not possible to successfully sample all calves at week 20. At this time point, 4 calves were sampled from the Control group, and 8 calves were sampled from both the Mixed and Dam groups.

To describe the microbial composition using molecular biology tools, 1 mL of rumen fluid was transferred to 2 mL Eppendorf tubes directly after sampling and centrifuged at 14,000 × g for 10 min at 4°C at the experimental farm. Supernatants were discarded and pellets were snap frozen in liquid nitrogen, transported to the laboratory and stored at −20°C until DNA extraction.

For VFA analysis, 0.8 mL rumen fluid filtrate was mixed with 0.5 mL of 4 mg/mL crotonic acid and 20 mg/mL metaphosphoric acid in 0.5 M HCl and frozen at −20 °C until analysis. VFA samples were centrifuged (16,500 × g, 10 min, 4°C) and the supernatant was analyzed by gas chromatography using crotonic acid as the internal standard on a Perkin Elmer Clarus 580 GC (Perkin Elmer, Courtaboeuf, France) equipped with a Stabilwax-DA column (30 m by 0.53 mm i.d.) (23). Each VFA sample was analyzed in duplicate. Samples for VFA at week 20 were not collected due to sampling constraints of the animals.

For protozoal counting, rumen fluid samples were mixed with a 1:1 methyl green-formalin solution and stored in the dark at room temperature. Protozoa were counted by microscopy using a Neubauer chamber, log-transformed, and classified as small entodiniomorphs (<100 μm) or large entodiniomorphs (> 100 μm) or as isotrichs [*Dasytricha (Dasy)* or *Isotricha (Iso)]* (24). Samples for protozoa at week 20 were not collected due to sampling constraints of the animals.

### 2.3 Microbial DNA extraction and metataxonomic analysis

Total genomic DNA from rumen contents was extracted using DNeasy Power Soil Pro Kit (Qiagen, Courtaboeuf, France) according to the manufacturer's instruction. This kit uses a combination of bead-beating and chemical lysis to break down feed particles and release DNA while minimizing contamination from inhibitors by implementing an on-column purification. In the final step DNA was eluted in 100 ml of ultrapure molecular grade water and conserved at−20°C. DNA concentration and quality were determined spectrophotometrically by measuring the A260/A280 on a Nanodrop spectrophotometer (Nanodrop Lite, France). Genomic DNA, diluted to 30 ng/μL was sent to ADNid (Qualtech Group, France) for library preparation using 519F−806R primers and PCR conditions as previously described (25). The amplicons of expected size of 280 base pairs were sequenced on a MiSeq instrument using a MiSeq Reagent Kit v2 according to the manufacturer's instructions (Illumina Inc., San Diego, CA, USA). A rumen mock community composed of synthetic DNA sequences for the 16S rRNA gene in equimolar quantities of *Methanobrevibacter millerae* SM9; *Methanobrevibacter olleyae* YLM1; *Methanomassiliicoccales* strain ISO4-H5; *Ruminococcus albus* 7; *Prevotella ruminicola* 23; *Fibrobacter succinogenes* subsp. succinogenes S85; *Butyrivibrio fibrisolvens* ATCC 19171; *Clostridium aminophilum* F; *Selenomonas ruminantium* GA192; *Eubacterium ruminantium* GA195; *Lachnospira multipara* DSM 3073T; *Peptostreptococcus anaerobius* ATCC 27337; *Streptococcus bovis* B315; *Megasphaera elsdenii* DSM 20460; *Pseudobutyrivibrio ruminis* HUN009 and *Propionibacterium* sp. JV5 followed the same sequencing and data analysis pipeline. The taxonomic assignment of the sequences reflected mock composition and revealed that the observed relative abundances aligned with the expected values. This indicates that the sequencing and classification process accurately reflected the anticipated composition of the community, confirming the reliability of the method used.

Raw sequences were quality filtered, chimera removed, and assigned taxonomy using the Quantitative Insights into Microbial Ecology II (QIIME 2) (26) with default parameters [DADA2 (27), Cutadapt (28), VSEARCH (29)] and GreenGenes2 database (30). A phylogenetic tree information from ASV sequences was included in the annotation step [using MAFFT (31) and FastTree (32)].

### 2.4 Statistical analysis

All statistical analyses and graphs were performed using R statistical software (version 4.4.1) (33). A linear mixed effect (lmer function of package lmerTest version 3.1.3) was used to test the effects of groups (Control *vs*. Dam *vs*. Mixed), time [age 2 days (only for IgG) *vs*. wk 3 *vs*. wk 10 *vs*. wk 14 (only for ADG)] and their interaction, as well as the effects of breed and sex as fixed effects and calves within a group as random effects on ADG and IgG concentration. Post hoc tests were performed using Tukey's Honest significant difference [Tuckey HSD (34)] at the 95% confidence level.

The data that did not meet the assumption of normality and homogeneity of variance, including VFA concentration and protozoa counting, were analyzed using the Kruskal-Wallis test (35) and a comparison between medians was performed using the Dunn's test (36) (P adjusted Bonferroni) in R software (using the FSA version 0.9.5 package). The chi square (Chi^2^) test was used to compare the frequency of diarrhea and respiratory disorders in the different groups. All statistical differences were considered as significant when *P* ≤ 0.05.

For metataxonomic data analysis, ASV and metadata tables from QIIME2 (26) were uploaded into R to create a phyloseq object using the qiime2R (37) and phyloseq (version 1.48.0) (38) packages. Alpha diversity was calculated using the Richness, Simpson, Shannon and Faith's Phylogenetic Diversity (PD) indices (39–41), using the phyloseq package. Plotting of alpha-diversity was performed using the ggboxplot function of ggpubr (version 0.6.0) (42), and statistical analysis was performed using the non-parametric Kruskal-Wallis test with Kruskal.test of the stats package (version 4.4.1). To assess beta diversity, Bray-Curtis (43) distance metric was calculated at ASV level using the vegan R library (44), and analysis of similarities (ADONIS) was performed using the adonis 2 function of vegan (version 2.6.6.1). Pairwise ADONIS was performed using anosim.pairwise with veganEx (version 0.1.0). Betadisper analysis was also performed using the Betadisper function of the vegan library. The MicroViz package (version 0.12.4) was used to plot relative abundance at phylum and genus levels. A Venn diagram was constructed using the MicrobiotaProcess package (version 1.16.1), with get_vennlist and venn.diagram functions.

Subsequently, a differential abundance analysis (DAA) of microbial features was conducted, comparing the groups at each week of age. As proposed by Nearing et al. (45) three different statistical approaches, namely MaAslin 2 (version 1.18.0) (46), LinDA (47) and LEfSe (version 1.12.1) (48), were used to enhance the robustness of the DAA findings. All analyses were conducted with the default settings and employing the centered log-ratio (CLR) transformation. Microbial taxa exhibiting a q-value < 0.1 following false discovery rate correction with the Benjamini-Hochberg approach were regarded as significant.

## 3 Results

### 3.1 Sequencing results

Following filtering, denoising and removal of chimeras using DADA2 implemented in QIIME2 (Supplementary Table 4), a total of 4,486,397 high-quality reads were obtained. Only samples exceeding the rarefaction threshold of 15,000 reads were included in the subsequent analysis. The final analysis excluded singletons and doubletons. One sample from the Mixed calves group had fewer than 15,000 sequences at the 3-week time point and was excluded from the subsequent filtering step, reducing the group to seven biological units. Only Amplicon Sequence Variant (ASVs) that were present in at least 75% of the samples for each group and sampling time point were retained. In total, 2,006 ASVs were identified across 99 samples, with a median read count of 27,689. Sequence reads are available in the NCBI Sequence Read Archive under BioProject PRJNA 1,183,330 with the corresponding BioSample Accessions SUB14851828.

### 3.2 Animal performance and health status were only moderately affected by the rearing management

**Figure 1:**
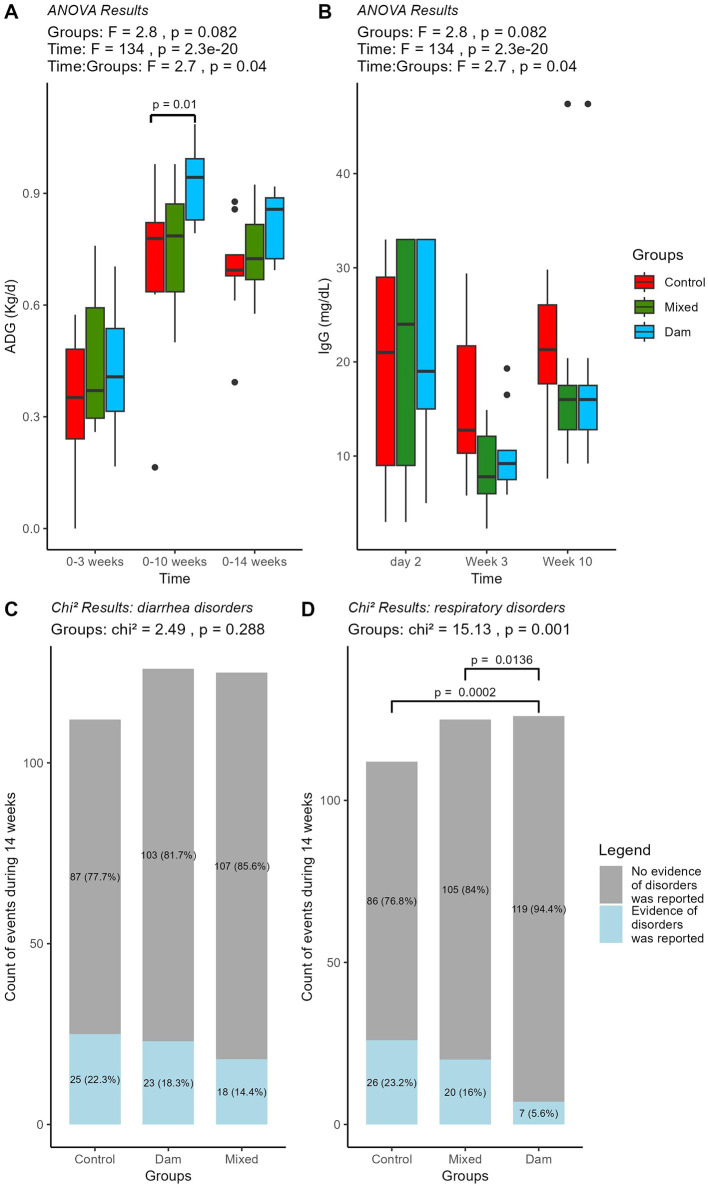
Performance and health status of calves separated from dams at birth (Control, *n* = 9), at the age of 4 weeks (Mixed, *n* = 9) or at weaning (Dam, *n* = 9). Boxplots representing median, upper and lower quartile for **(A)** Average daily gain (ADG) in kg per day and **(B)** Serum IgG concentration in mg per decilitre; blood was sampled at 2 days, 3 and 10 weeks of age. The results of the ANOVA test are shown at the top and significant *post-hoc* with TukeyHSD at 95% (*p* < 0.05) are shown in brackets. **(C)** Events of diarrhea and **(D)** respiratory disorders during 14 weeks in the three groups of calves. The gray box shows the number of calves with no health issues and the blue box shows all event in calves that were positive for disorder with the corresponding *P*-values.

Despite comparable birth weights, at 10 weeks of age, the Dam calves exhibited greater body weights (107.4 ± 7.23 kg) than those in the control group (92.2 ± 17.5 kg). This resulted in significant differences in average daily gain (ADG) at this time point, with a numerical difference persisting until week 14 (Figure 1A). No significant effect of breed or sex on ADG values was observed (Supplementary Table 5). The average IgG concentration at 2 days of age was (mean ± SD) 19.6 ± 11.3 mg/dL for Control, 19.3 ± 13.0 mg/dL for Mixed and 22.0 ±11.1 mg/dL for Dam. The IgG concentrations declined sligthly at 3 weeks, followed by an increase at the age of 10 weeks. However, the levels remained within a comparable range across all groups, indicating no significant interaction between time and groups (*P* > 0.05; Figure 1B, Supplementary Table 5). Although not statistically significant, the incidence of diarrhea episodes was numerically higher in the Control group (22.3%) than in the Dam (18.3%) and Mixed (14.4%) groups (Figure 1C). For respiratory disorders, the incidence was clearly lower in the Dam group (5.56%) than in the other two groups (*P* < 0.05; Figure 1D).

The total VFA concentration showed a comparable increase across all groups from 3 to 13 weeks (Table 1). At 10 weeks, the Mixed cohort displayed a higher total VFA concentration (*P* < 0.001) and a diminished proportion of propionate (*P* < 0.001) in comparison to the Dam and Control groups. At 13 weeks of age, the proportion of propionate in the Dam group was significantly higher (*P* < 0.001) compared to the other groups (Table 1).

**Table 1:** Rumen volatile fatty acids (VFA) concentration (mM) and relative proportions (mM/mM) in calves separated from dams at birth (Control), separated from dams at 4 weeks of age (Mixed) and separated from dams at weaning (Dam) and sampled at the age of 3, 10, and 13 weeks.

**Variables**	**Control**			**Mixed**			**Dam**			**IQR (interquartile range)**			***P*** **-value (Kruskal-Wallis)**		
	**wk 3**	**wk 10**	**wk 13**	**wk 3**	**wk 10**	**wk 13**	**wk 3**	**wk 10**	**wk 13**	**wk 3**	**wk 10**	**wk 13**	**Time**	**Groups**	**Groups (Time)^g^**
^h^Total VFA (mM)	40.9	62.4 ^b^	73.5	33.3	79.0 ^a^	71.7	36.9	41.6 ^b^	89.8	16.1	34.1	26.6	<0.001	0.55	<0.001
***VFA (%)***															
Acetate	78.4	70.0	70.5 ^ab^	80.2	71.9	71.8 ^a^	83.3	73.9	69.7^b^	4.46	4.89	2.06	<0.001	0.80	<0.001
Propionate	14.6	18.6 ^a^	17.2 ^c^	13.2	14.7 ^b^	17 ^c^	12.4	18.7 ^a^	20.6^d^	2.93	3.93	3.81	<0.001	0.18	<0.001
Butyrate	4.40	7.20 ^ab^	9.90	3.40	10.3 ^a^	9.30	2.80	5.30 ^b^	5.40	1.76	4.84	2.22	<0.001	0.02	<0.001
Valerate	0.60 ^a^	0.90 ^c^	0.70 ^ef^	0.40 ^ab^	0.60 ^d^	0.50 ^e^	0.30 ^b^	0.50 ^d^	0.80^f^	0.23	0.42	0.21	<0.001	<0.001	<0.001
Caproate	0.10 ^a^	0.07	0.09	0.04 ^ab^	0.08	0.1	0.02 ^b^	0.02	0.08	0.08	0.09	0.05	0.08	0.02	<0.001
Iso butyrate	0.90	0.70	0.50	0.80	0.50	0.50	0.80	0.70	0.50	0.31	0.36	0.18	0.003	0.30	0.05
Iso valerate	1.40	1.10	0.70	1.30	0.90	0.70	1.10	0.90	0.90	0.66	0.4.	0.23	<0.001	0.55	0.01

Values are medians for 9 observations per group.

g Time (Groups) is an artificial variable used to assess the nested effect of the Groups with only one category of the effect Time. When this effect was significant a Dunn *post-hoc* test with p-adjusted Benjamin-Hochberg Procedure (FDR) was applied. Different superscript letters in the same row and per sampling time show significant differences.

h Total VFA represent the sum of all individual VFAs.

### 3.3 Rumen microbial colonization dynamics are modulated by rearing practices

At the age of 3 weeks, Control calves had no rumen protozoa. In contrast, the presence of the dam (Dam and Mixed groups) facilitated the early colonization of the rumen by a diverse protozoal community (Table 2, Supplementary Figure 1). At the age of 10 weeks, the calves from the Mixed group had higher protozoa counts than the other two groups, and these levels remained elevated at the final sampling time. At 10 weeks of age, the control calves were the only group that did not demonstrate *Isotricha* colonization. However, at 13 weeks of age, the size of the *Isotricha* population was comparable between the three groups (Table 2, Supplementary Figure 1). The Control calves were the only ones that did not have *Dasytricha* during all sampling (Table 2, Supplementary Figure 1).

**Table 2:** Counts of protozoal groups in the rumen of calves separated from dams at birth (Control), separated from dams at 4 weeks of age (Mixed) and separated from dams at weaning (Dam) and sampled at the age of 3, 10, and 13 weeks.

**Variables**	**Control**			**Mixed**			**Dam**			**SEM**			***P*** **-value (Kruskal-Wallis)**		
	**wk 3**	**wk 10**	**wk 13**	**wk 3**	**wk 10**	**wk 13**	**wk 3**	**wk 10**	**wk 13**	**wk 3**	**wk 10**	**wk 13**	**Time**	**Groups**	**Time (Groups)^g^**
Protozoal numbers × 10^5^	0.00 ^a^	2.18^c^	2.18 ^e^	0.32 ^b^	4.18 ^d^	2.10 ^f^	0.40 ^b^	0.28 ^c^	3.75 ^e^	0.092	0.833	0.780	0.08	<0.001	<0.001
**Classification of protozoa**															
Small entodioniomorphs (< 100 μm) × 10^5^	0.00 ^a^	1.82 ^cd^	2.06	0.29 ^b^	3.67^c^	1.92	0.38 ^b^	0.22 ^d^	3.68	0.080	0.780	0.720	<0.001	0.004	<0.001
Large entodioniomorphs (> 100 μm) × 10^3^	0.00 ^a^	35.6	8.39 ^a^	2.80 ^b^	36.5	7.55 ^a^	2.32 ^b^	3.07	0.34 ^b^	0.530	10.30	1.490	0.02	0.004	<0.001
*Isotricha* × 10^3^	0.00	0.09 ^a^	3.80	0.00	13.6 ^b^	7.51	0.02	2.67 ^ab^	3.78	0.007	2.660	1.150	<0.001	0.02	<0.001
*Dasytricha* × 10^3^	0.00	0.01 ^a^	0.22	0.01	1.31 ^b^	3.05	0.01	0.55 ^ab^	2.80	0.003	0.307	1.030	0.002	0.06	0.002

Values are means for 9 observations per group; statistical analysis was performed on log-transformed values.

g Time (Groups) is an artificial variable used to assess the nested effect of the Groups with only one category of the effect Time. When this effect was significant a Dunn *post-hoc* test with p-adjusted Benjamin-Hochberg Procedure (FDR) was applied. Different superscript letters in the same row and per sampling time show significant differences.

**Figure 2:**
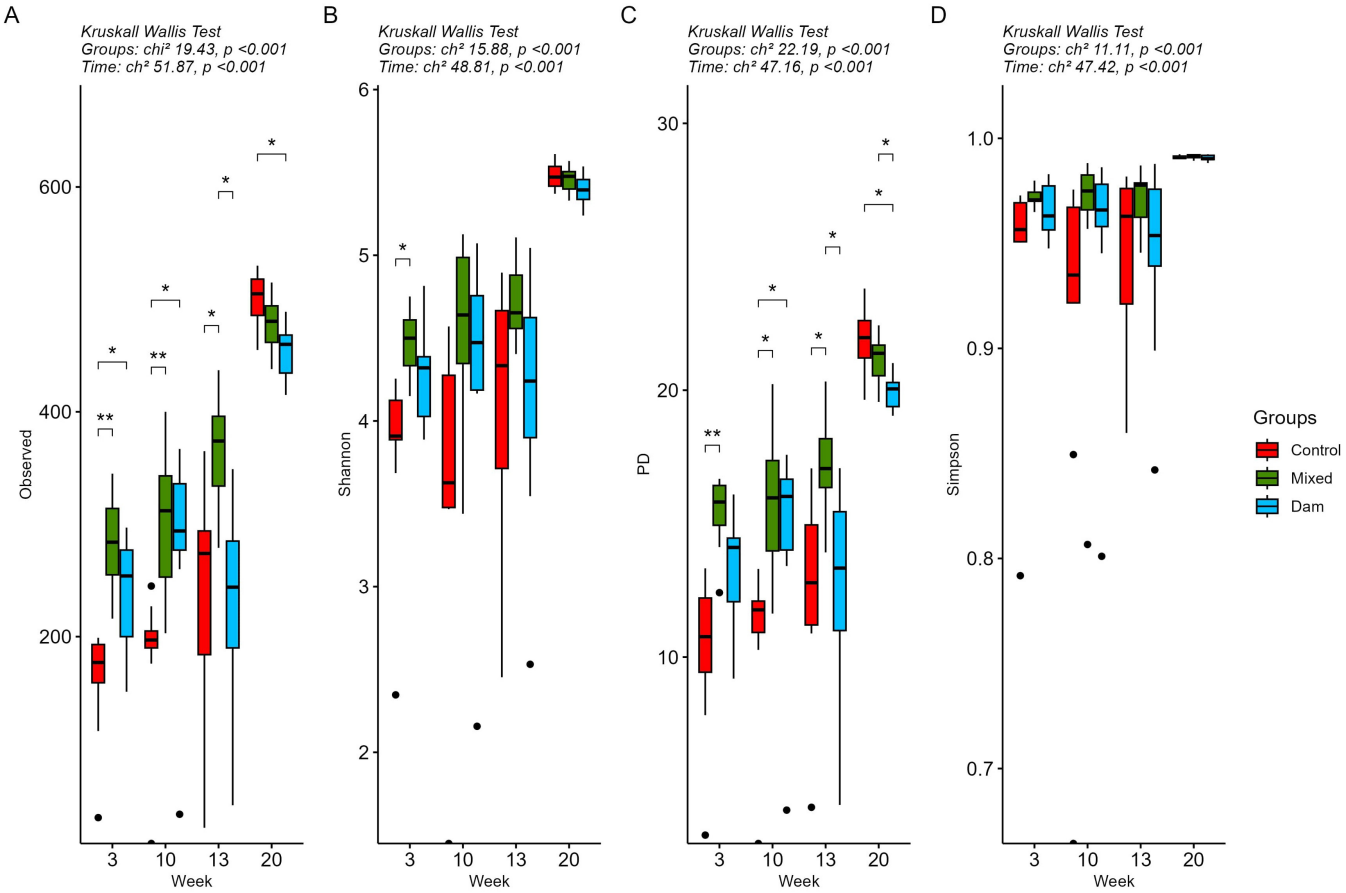
Alpha diversity analysis of rumen prokaryote (bacterial and archaea) community of calves separated from dams at birth (Control), at the age of 4 weeks (Mixed) or at weaning (Dam) and sampled at the age of 3, 10, 13 and 20 weeks. All calves were weaned at 11 weeks of age. Alpha diversity was measured using **(A)** Observed, **(B)** Shannon, **(C)** Phylogeny-based and **(D)** Simpson metrics. Statistical analysis was performed using the Kurskall Wallis test and outputs are displayed at the top of each graph. Asterisks and brackets indicate significant differences between groups within a time point (*P* < 0.05) determined using the p-adjusted Benjamini-Hochberg Procedure (FDR) reported by the Dunn *post-hoc* test.

For the prokaryotic (bacterial and archaeal) community, as the calves aged, an increase was observed in the values for all alpha diversity metrics (*P* < 0.001; Figure 2, Supplementary Table 6). At 3 weeks of age, all alpha diversity metrics were lower in the Control calves compared to those reared with the dams. However, these differences were only statistically significant in the comparison between the Control and Mixed groups, except for the Shannon index. This is consistent with more than 30% of ASVs being shared between the Dam and Mixed groups, while only 17% of them were also found in the Control group (Supplementary Figure 2). Alpha diversity indices showed group-specific fluctuations over time, but they converged toward the end of the study during the grazing period. Similarly, 2 weeks after weaning, the number of common ASVs in the three groups almost doubled compared to the two previous sampling periods, exceeding 40% at week 20 (Supplementary Figure 2).

**Figure 3:**
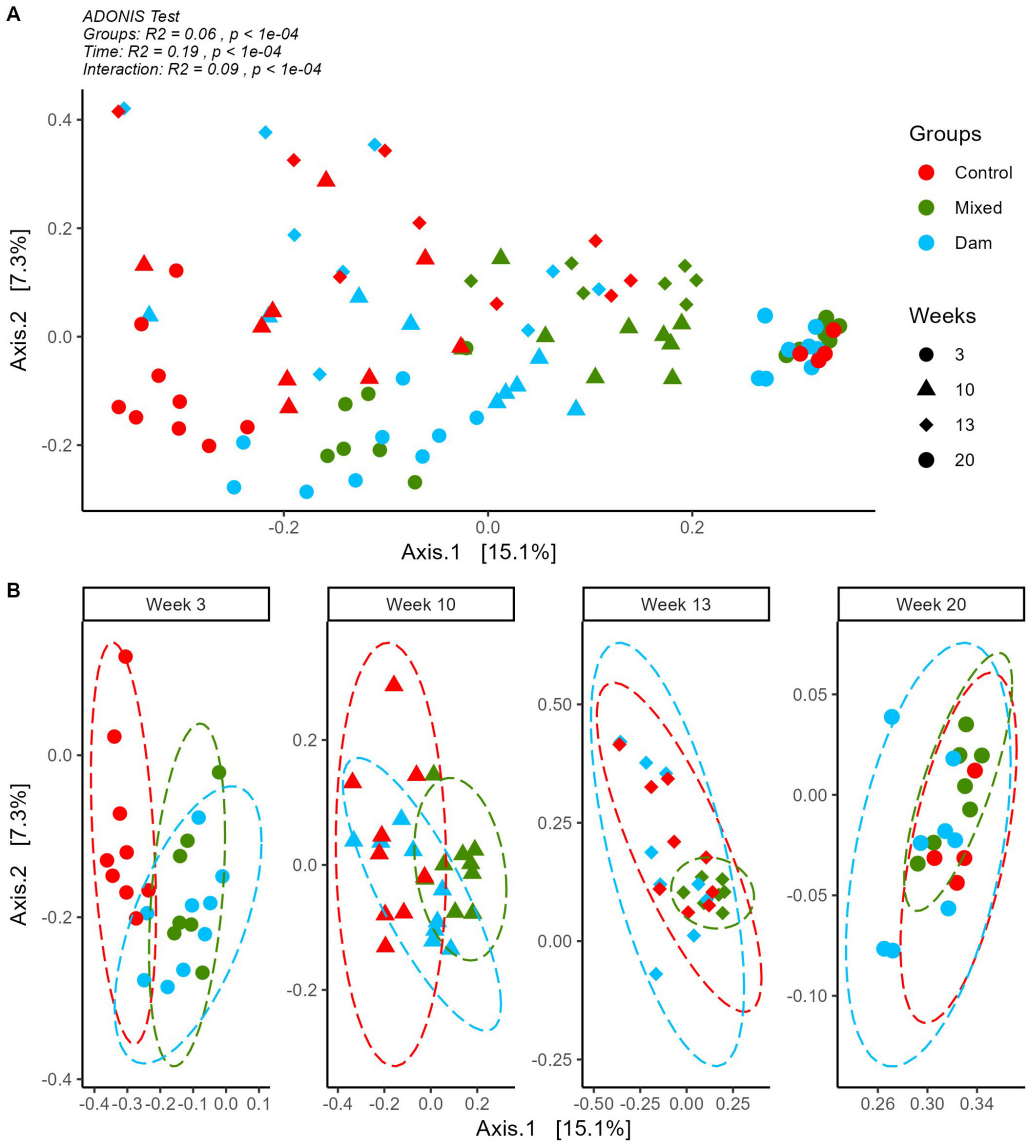
Principal coordinates analysis on Bray Curtis distances computed on ASV tables for sequences detected in rumen contents of calves separated from dams at birth (Control), at the age of 4 weeks (Mixed) or at weaning (Dam) and sampled at the age of 3, 10, 13 and 20 weeks. **(A)** Whole data set and ADONIS output for differences between Groups, Time and their interaction. **(B)** By week plotting of each group for easier visualization. Ellipses show group distribution at 95% confidence interval.

Temporal shifts in the prokaryote community structure of the calves were indicated by the PCoA plot and confirmed by the ADONIS test (time effect *R*^2^ = 0.19; *P* < 0.001; Figure 3A). Moreover, microbial community were also influenced by the Groups effect (group effect *R*^2^ = 0.06; *P* < 0.001) and a Time^*^Groups interaction was observed (interaction effect *R*^2^ = 0.09; *P* < *0.001*). In contrast, Breed (*R*^2^ = 0.01; *P* = *0.09*) and Sex (*R*^2^ = 0.02; *P* = *0.06*) were not significant.

Figure 3B shows that at 3 weeks of age, calves from the Dam and Mixed groups had similar microbial profiles that differed from the one of the Control group. This was also confirmed by the non-significant pairwise ADONIS comparison for Mixed and Dam groups (Supplementary Table 6). At 10 weeks all pairwise comparisons showed significant difference between the groups (Supplementary Table 7) and these differences persisted after weaning at week 13. At week 20, differences were still statistically significant (Supplementary Table 7), although these differences were barely discernible in the clustering (Figure 3B).

### 3.4 Cow transfer of protective microbiota can also be a source of pathogens for calves

The taxonomic profiles of rumen prokaryotes was represented by 14 phyla, with a dominance of Bacteroidota (formerly *Bacteroidetes*, 50.8%), followed by *Bacillota* (formerly *Firmicutes* A, 17.6%) and *Pseudomonadota* (formerly *Proteobacteria*, 10.8%). A total of 53 genera were found, with a predominance of *Prevotella* (26.1%) followed by *RF16* (18.5%; Supplementary Table 7) Relative abundance of phyla (Supplementary Figure 3A) and genera (Supplementary Figure 3B) varied modestly (Supplementary Table 7) between groups of the same age.

To describe age-related variations in the rumen microbial composition of growing calves, the search for discriminant microbial features was conducted independently for each sampling time point. At 3 weeks of age, microbial characteristics related to *Bacillus A* distinguished the Mixed and Dam groups from the control (*q* < 0.06 for LinDa, MaAslin and LEfSe), with a remarkable 10-fold higher relative abundance (Table 3). At 10 weeks of age, the lower abundance of opportunistic pathogens belonging to the genus *Tatumella* (*q* = 0.01 for LinDa and MaAslin) of the family *Erwiniaceae* differentiated the Dam and Mixed groups from the Control. In contrast, *Shewanella* (*q* = 0.01 for LinDa, *q* = 0.07 for MaAslin, *q* = 0.03 LEfSe), another opportunistic pathogen, was more abundant in Dam calves, distinguishing them from Mixed calves (Table 3). A large number of microbes discriminating between Dam and Mixed groups at 13 weeks of age were detected. These were typical rumen bacteria found in adult ruminants, such as *Prevotella* (*q* < 0.001 for LinDa and *q* = 0.07 for MaAslin), *Ruminococcus* (*q* < 0.01 for LinDa and MaAslin) or *Veillonnella* (*q* < 0.01 for LinDa and MaAslin), which were more abundant in calves that remained with their dams until weaning compared to those separated from their dams at 4 weeks of age, but this relationship was not found in control calves compared to either of the other two groups (Table 3). On the other hand, *Clostridia*, which was more abundant in the Control and Dam groups (*q* < 0.02 for LinDa, MaAslin and LEfSe), albeit at low levels, distinguished them from the Mixed calves. At 20 weeks of age, there were few discriminant microbes between the Dam and Mixed groups and these were low in abundance.

**Table 3:** Discriminant microbial features identified in the rumen of calves separated from dams at birth (Control, *n* = 9 for 3, 10, 13 and *n* = 4 for 20 weeks), at the age of 4 weeks (Mixed, *n* = 7 for 3 *n* = 10 and 13 and *n* = 8 for 20 weeks) or at weaning (Dam, *n* = 9 for 3, 10, 13 and *n* = 8 for 20 weeks) and sampled at the age of 3, 10, 13, and 20 weeks.

***Age (wk)***	**Pairwise comparaison**		**Family**	**Genus**	**Relative abundance**		**Method q value^1^**		
	**Group 1**	**Group 2**			**Group 1**	**Group 2**	**LinDa**	**MaAslin**	**LEfSe**
3	Mixed	Control	*Bacillaceae*	*Bacillus (A)*	0.18%	0.02%	0.01	0.06	0.01
3	Dam	Control	*Bacillaceae*	*Bacillus (A)*	0.20%	0.02%	0.01	0.03	NS
3	Dam	Control	*Bacillaceae*	*Baciilus (P)*	1.91%	1.30%	0.05	0.03	0.05
3	Mixed	Control	*Burkholderiaceae*	*Pusillimonas*	0.05%	0.00%	0.01	0.09	NS
10	Dam	Mixed	*Moraxellaceae*	*Acinetobacter*	0.04%	0.00%	0.02	0.09	NS
10	Mixed	Control		*Bacteroidales Order*	3.20%	0.56%	0.06	0.07	NS
10	Dam	Control		*Gammaproteobacteria Class*	0.58%	1.78%	0.06	0.05	NS
10	Dam	Mixed	*Micrococcaceae*	*Pseudarthrobacter*	0.07%	0.00%	0.004	0.03	NS
10	Dam	Mixed	*Shewanellaceae*	*Shewanella*	2.14%	0.54%	0.01	0.07	0.03
10	Dam	Control	*Erwiniaceae*	*Tatumella*	0.00%	0.34%	0.01	0.01	NS
10	Mixed	Control	*Erwiniaceae*	*Tatumella*	0.00%	0.34%	0.01	0.01	NS
13	Dam	Mixed		*Bacillales Order*	0.03%	0.52%	<0.001	<0.001	NS
13	Dam	Control		*Bacillales Order*	0.03%	0.37%	0.001	0.001	NS
13	Dam	Mixed		*Bacilli Class*	4.29%	2.70%	<0.001	0.03	NS
13	Dam	Control	*Bacillaceae*	*Bacillus (A)*	0.00%	0.04%	NS	0.07	NS
13	Mixed	Control		*Bacteroidales Order*	3.32%	0.51%	0.02	0.07	NS
13	Dam	Mixed		*Bacteroidia Class*	0.00%	0.01%	<0.001	0.005	NS
13	Dam	Mixed		*Clostridia Class*	4.11%	2.74%	<0.001	0.06	NS
13	Control	Mixed	*Peptostreptococcaceae*	*Clostridioides*	0.55%	0.01%	0.02	0.005	0.02
13	Dam	Mixed	*Peptostreptococcaceae*	*Clostridioides*	0.10%	0.01%	<0.001	0.007	NS
13	Mixed	Control	*DTJY01*	*DTJY01*	0.15%	0.06%	0.02	0.08	NS
13	Mixed	Control		*Enterobacterales Order*	0.04%	0.00%	0.02	0.09	NS
13	Dam	Mixed	*Bacteroidaceae*	*Phocaeicola*	3.55%	0.79%	0.001	0.04	0.003
13	Dam	Mixed	*Bacteroidaceae*	*Prevotella*	34.6%	31.5%	<0.001	0.07	NS
13	Dam	Mixed	*Micrococcaceae*	*Pseudarthrobacter*	0.00%	0.04%	0.008	0.005	NS
13	Dam	Mixed	*Pseudomonadaceae*	*Pseudomonas*	0.03%	0.00%	<0.001	0.005	NS
13	Control	Mixed	*Oscillospiraceae*	*Ruminococcus*	10.67%	7.02%	NS	0.03	0.08
13	Dam	Mixed	*Oscillospiraceae*	*Ruminococcus*	9.80%	7.02%	<0.001	0.007	NS
13	Dam	Mixed	*Shewanellaceae*	*Shewanella*	0.00%	0.20%	0.03	0.02	0.03
13	Dam	Mixed	*Veillonellaceae*	*Veillonella*	5.64%	2.79%	<0.001	0.008	0.05
20	Dam	Mixed		*Oscillospirales Order*	0.02%	0.15%	0.02	0.05	NS
20	Dam	Mixed	*Bacteroidaceae*	*Phocaeicola*	1.37%	0.52%	0.005	0.006	0.06
20	Dam	Mixed	*Rhizobiaceae*		0.00%	0.08%	0.004	0.01	NS
20	Dam	Mixed	*Streptomycetaceae*	*Streptomyces*	1.37%	0.09%	0.004	0.01	NS
20	Dam	Mixed	*Acutalibacteraceae*	*CAG-488*	2.65%	5.02%	0.001	0.004	NS

Discriminant analysis was performed using LinDA, MaAslin and LefSe and all presented features below were identified as discriminant by at least two of the three methods used.

1 False discovery rate (Benjamini-Hochberg) correction.

## 4 Discussion

The aim of this study was to determine the effects of early and late maternal separation of calves and to evaluate the potential benefits of an intermediate 4-week period of cow-calf contact on animal growth performance, health and rumen microbial acquisition. We found a higher ADG in the Dam group before weaning and a lower incidence of respiratory disease compared to the Mixed and Control groups. However, these differences, that can be considered minor, are in stark contrast to the high variation observed for the microbial colonization of the rumen. It is noted that these differences in colonization may be due to intrinsic, confounding changes in husbandry and feed management practices, such as that the Dam calves had access to pasture a few days after birth as they followed their dams out of the barn. Of particular interest is the observation that opportunistic pathogens were predominantly present in the rumen of calves separated from their dams at birth, whereas later in life pathogenic strains were specifically identified in calves that remained with their mothers until weaning.

It was expected that the growth rate of calves reared with dams would be improved due to the expected higher milk consumption. Long-term contact with the mothers improved calf growth at the end of the suckling period in the mother group. Indeed, after the removal of the males at the end of the third week, the nine calves remaining in the mothers' group had better access to milk due to their proximity to other cows. The cow-calf behavior study by Nicolao (49) showed that Holstein cows in particular were accepting to nurse other calves (49). However, the effect on growth was not observed in the mixed group, which had 4 weeks of contact, potentially due to the stress associated with separation from the mother and adaptation to the automatic milk feeder. In the same behavioral study by Nicolao (49), an increase in vocalization was seen as a sign of stress in this group. No statistically significant differences in body weight were observed between groups at 14 weeks of age. The phenomenon of increased vocalization was observed in all groups around 1 week after weaning (49), showing a sign of stress that may explain similar weights between groups at 14 weeks of age. The results are in line with those of other researchers who identified a difference in body weight between calves in full contact with the mother and those without during their first seven weeks of life. However, this difference was not maintained until the calves reached 6 months of age (50). Similarly, artificially and naturally reared lambs have been shown to reach the same weight at the end of the grazing period (51).

The concentration of IgG was found to be identical in all groups at 2 days of age, indicating a uniform passive transfer of immunity. The transfer of IgG is of particular importance in the prevention of infectious diseases in calves (52). Furthermore, the comparable concentration of IgG throughout the experimental period is consistent with the absence of any discernible difference in the symptoms of diarrhea (53). The incidence of symptoms of respiratory disorders was lower in the Dam group than in the Control group.

The rumen microbial structure was significantly influenced by rearing practices, which encompass contact with adult animals and feeding management. In this study, calves from each group were housed in separate pens, but all were located within the same barn. Thus, we assumed that the environment was the same and that the main differences were due to contact or lack of contact with the dam, and that the resulting differences in milk and solid feed intake were an inherent confounding factor. The main factor explaining the variance was the age of calves (Time) that accounted for ~20%, followed by rearing practices (feed and presence of mother) that explained a further 6%. Both are considered determinist factors for the establishment of the rumen microbiome (54). Breed is another factor able to modify the rumen microbiome but it was not significant in our study. However, there may be also other factors that can influence the the rumen microbiota that were not accounted for in this study. As early as 3 weeks of age, the rumen microbial composition differed between maternal and artificial rearing, in accordance with previous studies with lambs (11) and goats (55). In calves, the research conducted by Beaver et al. (9) indicated that the presence of the mother was associated with a variation in the fecal microbiota. The similarity in rearing conditions in the first weeks of life for calves in the Dam and Mixed groups was reflected in the almost one-third shared ASVs between these groups in week 3, though Mixed calves had no access to pasture. In contrast, for the same period <20% of the ASVs were shared between Control and Mixed or Dam groups.

Similarly, rumen protozoa were detected before weaning exclusively in these two dam-reared groups (Dam and Mixed groups). Protozoa colonization is possible through direct contact with adults (12) and their presence may explain the differences observed in the bacterial community (56). A hypothesis has been put forth suggesting that bacterial predation by protozoa may serve to stimulate the proliferation of different bacterial species that occupy similar metabolic niches (57). In agreement, the rumen microbiota of the Mixed and Dam calves at the age of 10 weeks exhibited a greater number of species, accompanied by higher Shannon and Simpson diversity indexes, which consider both species richness and evenness. These differences were maintained at the age of 10 weeks even though Mixed calves were already separated from their mothers for 6 weeks. We also noted that the Mixed group appeared to be less dispersed than the other two groups at 10 and 13 weeks of age. It has been shown that an early introduction of solid feed allows a rapid shift in rumen β-diversity followed by stabilization, whereas a late introduction of solid feed results in a gradual transition in β-diversity (10). Separation after 4 weeks in the Mixed group probably induced an increase in solid feed intake and reduced individual differences in β-diversity within the group. These findings indicate that a period of up to 4 weeks of cow-calf contact is sufficient for the acquisition and subsequent maintenance of a diverse microbial community in the rumen.

The variability observed during the pre-weaning and early post-weaning periods (at 10 and 13 weeks of age) can be attributed to colonization sequential dynamics driven by the diet transition. It has been reported that amylolytic bacteria are more prevalent in concentrate-fed calves than in those fed on pasture (58). In our study of the early post-weaning phase, the discriminant genera between the Dam and Mixed groups were predominantly cellulolytic, including *Ruminococcus* and lactate-using bacteria such as *Veillonella*. Additionally, the highly versatile carbohydrate-using bacteria *Prevotella* were also identified. We speculate that the two stressful periods to which the Mixed group of calves were subjected may have affected the proportion of these predominant microbial taxa in the mature rumen. This is in contrast to the Dam group, which showed higher proportion of *Prevotella spp*. that could be due to the early access to pasture. It is noteworthy that these bacterial taxa did not discriminate the Control group. This can be attributed to the limited sample size, which hindered the ability to adequately capture the high variability associated with stochastic colonization. Although stochastic events would decrease after 3 weeks of age, stabilization of the rumen microbiota is reported to occur after 3 months of age (59, 60). Moreover, the absence of the mother as the primary source of microbial transmission (8) further affected microbial colonization in the animals. This highlights the importance of including a larger cohort and considering maternal influence when studying microbial communities in early-life stages.

By the final sampling time at 20 weeks, there was a notable reduction in inter-animal variability, indicating a stabilization of the rumen microbiota, which is likely attributable to the maturity of the rumen environment due to the age of the animals and the longer time since the transition to a solid diet compared to earlier sampling times. It is well documented in the literature that diet plays a pivotal role in shaping the rumen microbial community in ruminants (61–63). Thus, the high degree of similarity between the rumen microbial community structures of calves from the three groups at the final sampling point at 20 weeks, when they were all grazing but in different paddocks and had no contact with other calves, is to be regarded as an likely outcome. Nevertheless, a persistent group effect was identified which can be attributed to the core successional microbes, the first microbes to colonize the rumen, which persist longer than other community members (59). The maintenance of discrepancies between groups has been documented in numerous investigations in early life modulation, albeit in disparate microbial communities. In lambs, changes were observed exclusively in the protozoa (11), a finding that aligns with our study up to 2 weeks post-weaning. Additionally, the rumen bacterial community was found to differ with the administration of different solid feeds prior to weaning, and this difference persisted until the animal reached one year of age (63). The evidence indicates that rumen microbial stabilization in post-weaned ruminants necessitates a prolonged time frame, potentially exceeding the 20-week duration of our study. However, further inquiry into the dynamics of convergence toward a final steady state is recommended, given that the immature rumen is conducive to microbial modifications aimed at optimizing the functioning of the ecosystem (64).

In order to ascertain microbial characteristics that could differentiate between calves in disparate groups at each sampling interval, a variety of statistical tools were employed. The microbiota of calves in contact with their dams (Dam and Mixed groups) at the age of 3 weeks was found to be characterized by a higher abundance of Bacillus-related taxa. Dietary supplementation with *B. subtilis* (65) or *B. megaterium* (66) has been shown to improve growth performance and reduce the incidence of diarrhea in calves. It has also been suggested that *B. subtilis* may facilitate the establishment of beneficial rumen microbes, thereby improving post-weaning feed efficiency (67). In the present case, the higher abundance of Bacillus-related taxa does not appear to be associated with protection against symptoms of diarrhea. It can be hypothesized that the presence of a potential pathogen may modify the ecosystem, with Bacillus-related taxa being associated with other microbes that do not offer protection against diarrhea in the Dam group. Furthermore, the lower prevalence of the opportunistic pathogen *Tatumella* in the Dam and Mixed groups at 3 weeks supports the notion that maternal transmission of microbiota plays a protective role. However, our results also suggest that prolonged cow-calf contact may facilitate the colonization by opportunistic pathogens. Indeed, at 10 weeks of age, the Dam calves exhibited a higher abundance of *Shewanella*-related species compared to the other groups, while at 13 weeks of age, the Mixed calves had a lower abundance of *Clostridioides*. *Shewanella* is a human pathogen (68), but they are rarely isolated from cattle (69) and, to our knowledge, no clinical cases have been reported in ruminants. *Shewanella* are a genus of Gram-negative bacteria that are commonly found in marine and soil environments, but they are rarely isolated from cattle (69). The hypothesis that an increase in the proportion of some potential bacterial pathogens is supported by the prolonged cow-calf contact observed in our study is therefore worthy of further investigation.

## 5 Conclusion

The present study demonstrated that 4 weeks of cow-calf contact is sufficient for the transfer of a well-diversified and potentially protective rumen microbial community. Maternal presence had no effect on body weight at the end of the study and modulated potential pathogens in either direction (increase or decrease). However, further confirmation of these results is recommended with larger numbers of animals and additional sampling (longer evaluation period) and parameters investigated (e.g., effect on first lactation) to determine the optimal postpartum cow-calf bonding period for enhanced microbial transmission, improved health, performance and animal welfare.

## Data Availability

The datasets presented in this study can be found in online repositories. The names of the repository/repositories and accession number(s) can be found at: https://www.ncbi.nlm.nih.gov/, BioProject PRJNA1183330.
