# Supplementary material for: Duration of dam contact had a long effect on calf rumen microbiota without affecting growth
Source: Front Vet Sci. 2025 May 12;12:1548892. doi: 10.3389/fvets.2025.1548892 (PMC12105649; doi:10.3389/fvets.2025.1548892)
Supplement: Supplementary file 1 [file Data_Sheet_1.docx]

Supplementary Material

# Supplementary Tables

**Supplementary Table 1. Description of the experimental groups**

|  | | Control ^1^ | Mixed^1^ | Dam^1^ |
| --- | --- | --- | --- | --- |
| Gender | ♀ | 6 | 8 | 8 |
|  | ♂ | 3 | 1 | 1 |
| Breed | Holstein | 5 | 5 | 6 |
|  | Montebeliard | 4 | 4 | 3^2^ |

^1^ Control (separated from dams at birth), Mixed (separated from dams at 4 weeks of age) and Dam (separated from dams at weaning)

^2^ One calf was Holstein-Montebeliard crossbred.

**Supplementary Table 2**. Feeding schedules for the calves in Control (separated from dams at birth), Mixed (separated from dams at 4 weeks of age) and Dam (separated from dams at weaning) groups.

| Period | Age (Weeks) | Control | | | Dam | | | Mixed | | |
| --- | --- | --- | --- | --- | --- | --- | --- | --- | --- | --- |
|  |  | Milk (Kg/d)^1^ | Concentrate (Kg/d)^2^ | Hay (Kg/d)^3^ | Milk (Kg/d)^1^ | Concentrate (Kg/d)^2^ | Hay (Kg/d)^3^ | Milk (Kg/d)^1^ | Concentrate (Kg/d)^2^ | Hay (Kg/d)^3^ |
| Birth to weaning | 1 | 6 | 0 | 0 | 24/24 | 0 | 0 | 24/24 | 0 | 0 |
|  | 2 | 7 | 0 | 0.5 | *ad libitum* suckling^4^ | 15:30 to 9:00  *ad libitum* | | *ad libitum* suckling^4^ | 15:30 to 9:00  *ad libitum* | |
|  | 3 | 8.5 | 0.2 |  |  |  |  |  |  |  |
|  | 4 | 10 | 0.4 |  |  |  |  | 10 | 0.4 | 0.5 |
|  | 5 | 10 | 0.6 |  |  |  |  | 10 | 0.6 |  |
|  | 6 | 10 | 0.9 |  |  |  |  | 10 | 0.9 |  |
|  | 7 | 9 | 1.2 |  |  |  |  | 9 | 1.2 |  |
|  | 8 | 7 | 1.5 |  |  |  |  | 7 | 1.5 |  |
|  | 9 | 5 | 1.8 |  |  |  |  | 6 | 1.8 |  |
|  | 10 | 5 | 2 |  |  |  |  | 5 | 2 |  |
| Weaning | 11 to 14 | 0 | 2 | *ad libitum* | 0 | 2 | *ad libitum* | 0 | 2 | *ad libitum* |
| Grazing ^5^ | 15 to 20 | 0 | 2 | 0.5 + grazing | 0 | 2 | 0.5 | 0 | 2 | 0.5 |

^1^ Automatic feeder dispenses bulk milk individually

^2^ First age concentrate distributed individually by automatic feeder (Control group and Mixed group after separation from the dam) or in collective bucket (Dam group and Mixed group before separation from the dam)

^3^ Hay from permanent grassland (first cut), distributed in a rack

^4^ 9:00 to 15:30 ad lib suckling with Dam

^5^ A permanent pasture divided into three adjacent, equal-sized plots of 0.15 hectares each. The pasture was primarily composed of *Lolium perenne* (39.0%), *Agrostis tenuis* (15.0%), and *Trifolium repens* (13.5%).

**Supplementary Table 3**.

Characteristics of vegetation offered on the experimental plots (mean ± standard deviation) as reported by (Nicolao et al., 2020)

| **Plot characteristics** | **Dam** | **Mixed** | **Standard** |
| --- | --- | --- | --- |
| Patch type (%) and description |  |  |  |
| Dry (≥70% dead material) | 15.5 ± 4.1 | 16.0 ± 2.1 | 13.3 ± 2.5 |
| Green (< 70% dead material) | 84.5 ± 7.2 | 84.0 ± 4.1 | 86.7 ± 6.0 |
| Grasses (≥ 70% grasses) | 65.1 ± 9.7 | 69.1 ± 6.6 | 64.2 ± 7.3 |
| Legumes (≥ 30% legumes) | 17.1 ± 4.2 | 13.6 ± 2.0 | 13.3 ± 3.4 |
| Forbs (≥ 30% forbs) | 17.8 ± 3.6 | 17.3 ± 1.4 | 22.5 ±4.8 |
| Tall (≥ 25 cm) | 51.2 ± 7.8 | 48.1 ± 4.5 | 53.3 ± 7.6 |
| Intermediate (7 cm ≤ x < 25 cm) | 33.3 ± 5.7 | 35.8 ± 4.1 | 32.5 ± 5.1 |
| Short (< 7 cm) | 15.5 ± 5.2 | 16.0 ± 2.6 | 14.2 ± 3.1 |
| Composition and nutritional value |  |  |  |
| Dry matter (g/kg) | 32.5 ± 3.3 | 28.3 ± 5.9 | 31.6 ± 2.5 |
| Organic matter digestibility (g/kg DM) | 67.2 ± 2.8 | 67.0 ± 3.8 | 66.0 ± 1.7 |
| NDF (g/kg DM) | 53.9 ± 4.4 | 53.8 ± 1.2 | 53.9 ± 1.8 |
| ADF (g/kg DM) | 27.6 ± 1.6 | 28.2 ± 1.0 | 28.5 ±1.9 |
| Crude protein (g/kg DM) | 12.0 ± 1.6 | 12.2 ± 2.7 | 10.6 ±1.8 |

**Supplementary Table 4**.

Bioinformatics metrics for metataxonomic analysis using QIIME 2 pipeline. Sequences for all experimental groups, Control (calves separated from dams at birth, Mixed (separated from dams at the age of 4 weeks) and Dam (separated from dams at weaning) and and all sampling times (wk 3, 10, 13 and 20) were analysed in the same bioinformatic run.

|  | Control | | | | Mixed | | | | Dam | | | |
| --- | --- | --- | --- | --- | --- | --- | --- | --- | --- | --- | --- | --- |
|  | wk 3 | wk 10 | wk 13 | wk 20 | wk 3 | wk 10 | wk 13 | wk 20 | wk 3 | wk 10 | wk 13 | wk 20 |
| **Input raw reads count** (mean ±SD) | 47559  (±8564) | 53574  (±4347) | 51816  (±3799) | 50043  (±4785) | 40887  (±14615) | 49999  (±5446) | 53489  (±4615) | 46834  (±5456) | 48126  (±6209) | 50052  (±4379) | 51009  (±1911) | 47001  (±4529) |
| **Filtered reads count** (mean ±SD)^1^ | 42077  (±8029) | 48102  (±4668) | 46451  (±4039) | 41611  (±3257) | 33843  (±13330) | 43136  (±4856) | 47626  (±4032) | 39125  (±4552) | 41064  (±5639) | 43448  (±4945) | 46190  (±2308) | 38565  (±3654) |
| **Filter data %** | 88  (±2) | 90  (±3) | 90  (±3) | 83  (±2) | 82  (±8) | 86  (±3) | 89  (±2) | 84  (±1) | 85  (±4) | 87  (±4) | 91  (±3) | 82  (±3) |
| **Denoised reads count** (mean ±SD) | 41087  (±7788) | 47063  (±4729) | 45264  (±4016) | 39769  (±3229) | 32896  (±13012) | 41654  (±5006) | 46161  (±4007) | 37200  (±4503) | 39789  (±5542) | 41954  (±5094) | 45106  (±2319) | 36717  (±3615) |
| **Merged reads count** (mean ±SD) ^2^ | 37956  (±7871) | 43155  (±5117) | 41222  (±3889) | 32988  (±2980) | 30052  (±11965) | 36695  (±5343) | 41158  (±3808) | 30559  (±3982) | 36136  (±5733) | 37376  (±5709) | 41100  (±3151) | 30427  (±3275) |
| **Merged data %** | 79  (±4) | 80  (±4) | 80  (±5) | 66  (±1) | 74  (±10) | 73  (±5) | 77  (±2) | 65  (±1) | 75  (±5) | 74  (±7) | 81  (±5) | 65  (±3) |
| **Non Chimeric reads count** (mean ±SD) ^3^ | 37720  (±7792) | 42665  (±5020) | 40956  (±3749) | 32942  (±2978) | 29971  (±11924) | 36576  (±5246) | 40980  (±3697) | 30504  (±3977) | 36012  (±5719) | 37126  (±5496) | 40922  (±3007) | 30357  (±3256) |
| **Non Chimeric data %** | 79  (±4) | 79  (±4) | 79  (±4) | 66  (±1) | 74  (±10) | 73  (±5) | 77  (±2) | 65  (±1) | 75  (±5) | 74  (±6) | 80  (±5) | 65  (±3) |

^1^Quality filtering removed all sequences with a Phred score <26 and length not comprised between 260 and 280 nucleotides min length 270 max length 276

^2^ Paired-end reads are merged, combining the forward and reverse reads into single sequences using the overlapping regions of on average 220 nucleotides

^3^chimera removal was performed using the UCHIME algorithm implemented in QIIME2

**Supplementary Table 5.** Average daily gain and plasma concentration igG in calves separated from dams at birth (Control), at the age of 4 weeks (Mixed) or at weaning (Dam) over the experimental period, from Day 2 to the age of 14 weeks. Data was analysed using Linear Mixed-effects Model of ADG and IgG matrices. R syntax for the final model is: lmer(values ~ Time + Group +sex + Breed+ Time * Group+(1 | id_animal), data = data).

| Groups | Control (n=9) | | | |  | Mixed (n=9) | | | | Dam (n=9) | | | | SEM | P value | | | | |
| --- | --- | --- | --- | --- | --- | --- | --- | --- | --- | --- | --- | --- | --- | --- | --- | --- | --- | --- | --- |
| Age | Day 2 | Wk 3 | Wk 10 | Wk 14 |  | Day 2 | Wk 3 | Wk 10 | Wk 14 | Day 2 | Wk 3 | Wk 10 | Wk 14 |  | Time | Group | Time*Group | Sex | Breed |
|  |  |  |  |  |  |  |  |  |  |  |  |  |  |  |  |  |  |  |  |
| ADG (kg / day) | NA | 0.34 | 0.70 ^a^ | 0.70 |  | NA | 0.45 | 0.75 ^ab^ | 0.73 | NA | 0.43 | 0.93 ^b^ | 0.81 | 0.027 | <0.001 | 0.08 | 0.04 | 0.14 | 0.72 |
| IgG (mg/dL) | 19.6 | 15.8 | 20.9 | NA |  | 19.3 | 8.60 | 18.3 | NA | 22.0 | 10.5 | 18.3 | NA | 1.152 | <0.001 | 0.47 | 0.62 | 0.41 | 0.30 |

^ab^ In the same age different letters show significant differences between groups reported by Tuckey HSD multiple comparison tests (p<0.05)

NA : Calves were not weighted at day 2; IgG was not measured in week 14

**Supplementary Table** **6**. Alpha diversity metrics computed using the phyloseq package on the rarefied dataset of microbial ASVs detected in the rumen content of calves separated from dams at birth (Control, n= 9 for 3, 10 and 13 weeks and n= 4 for 20 weeks), at the age of 4 weeks (Mixed, n= 7 for 3 weeks, n=9 for 10 and 13 weeks and, n= 8 for 20 weeks) or at weaning (Dam, n= 9 for 3,10 and13 weeks and, n= 8 for 20 weeks).

| Groups | Control | | | | Mixed | | | | Dam | | | | IQR (interquartile range) | | | | P value (Kruskall Wallis Test) | | |
| --- | --- | --- | --- | --- | --- | --- | --- | --- | --- | --- | --- | --- | --- | --- | --- | --- | --- | --- | --- |
| Age | wk3 | wk10 | wk13 | wk20 | wk3 | wk10 | wk13 | wk20 | wk3 | wk10 | wk13 | wk20 | wk3 | wk10 | wk13 | wk20 | Time | Group | Time(Groups)^1^ |
| Alpha diversity index | | |  |  |  |  |  |  |  |  |  |  |  |  |  |  |  |  |  |
| Observed | 177^a^ | 197^c^ | 274^e^ | 505^g^ | 284^b^ | 312^d^ | 374^f^ | 480^gh^ | 254^b^ | 294^d^ | 244^e^ | 460^h^ | 90.0 | 121 | 125 | 43.0 | <0.001 | <0.001 | <0.001 |
| *PD* | 10.8^a^ | 11.8^c^ | 12.8^e^ | 22.0^g^ | 15.8^b^ | 16.0^d^ | 17.1^f^ | 21.4^g^ | 14.1^ab^ | 16.0^d^ | 13.3^ef^ | 20.1^h^ | 4.35 | 4.45 | 4.96 | 1.71 | <0.001 | <0.001 | <0.001 |
| Shannon | 3.91^a^ | 3.63 | 4.33 | 5.47 | 4.50^b^ | 4.64 | 4.65 | 5.48 | 4.32^ab^ | 4.47 | 4.24 | 5.39 | 0.49 | 0.84 | 0.75 | 0.13 | <0.001 | <0.001 | <0.001 |
| Simpson | 0.96 | 0.93 | 0.96 | 0.99 | 0.97 | 0.97 | 0.98 | 0.99 | 0.96 | 0.97 | 0.95 | 0.99 | 0.02 | 0.04 | 0.04 | 0.00 | <0.001 | <0.001 | <0.001 |

^1^ Time(Groups) is an artificial variable used to assess the nested effect of the Groups with only one category of the effect Time.

^abcdefgh^ In the same age different letters show significant differences between groups reported by Dunn post-hoc test with p-adjusted Benjamin-Hochberg Procedure (FDR)

**Supplementary Table 7.** Pairwise multilevel comparison using ADONIS and BETADISPER on Bray-Curtis distance matrices calculated from ASV rarefied tables for calves separated from their mothers at birth (Control, n= 9 for 3, 10 and 13 weeks and, n= 4 for 20 weeks), at the age of 4 weeks (Mixed, n= 7 for 3 weeks, n=9 for 10 and 13 weeks and, n= 8 for 20 weeks) or at weaning (Dam, n= 9 for 3, 10 and13 weeks and, n= 8 for 20 weeks).

|  |  | Control *vs.* Dam | | | Control *vs.* Mixed | | | Mixed *vs.* Dam | | |
| --- | --- | --- | --- | --- | --- | --- | --- | --- | --- | --- |
|  | | Adonis | | Betadisper | Adonis | | Betadisper | Adonis | | Betadisper |
| Sampling time | | R | *P* adjusted | *P* values | R | *P* adjusted | *P* values | R | *P* adjusted | *P* values |
| wk 3 | | 0.15 | 0.002 | 0.05 | 0.16 | 0.002 | 0.08 | 0.07 | 0.19 | 0.71 |
| wk 10 | | 0.13 | 0.001 | 0.35 | 0.17 | 0.001 | 0.15 | 0.15 | 0.001 | 0.70 |
| wk 13 | | 0.09 | 0.04 | 0.93 | 0.15 | 0.003 | 0.09 | 0.16 | 0.003 | 0.06 |
| wk 20 | | 0.14 | 0.01 | 0.06 | 0.15 | 0.006 | 0.28 | 0.17 | 0.003 | 0.22 |

# Supplementary Figures

**Supplementary Figure 1**. Median and interquartile ranges of rumen protozoan communities of calves separated from dams at birth (Control, n= 9), at the age of 4 weeks (Mixed, n= 9) or at weaning (Dam, n= 9).


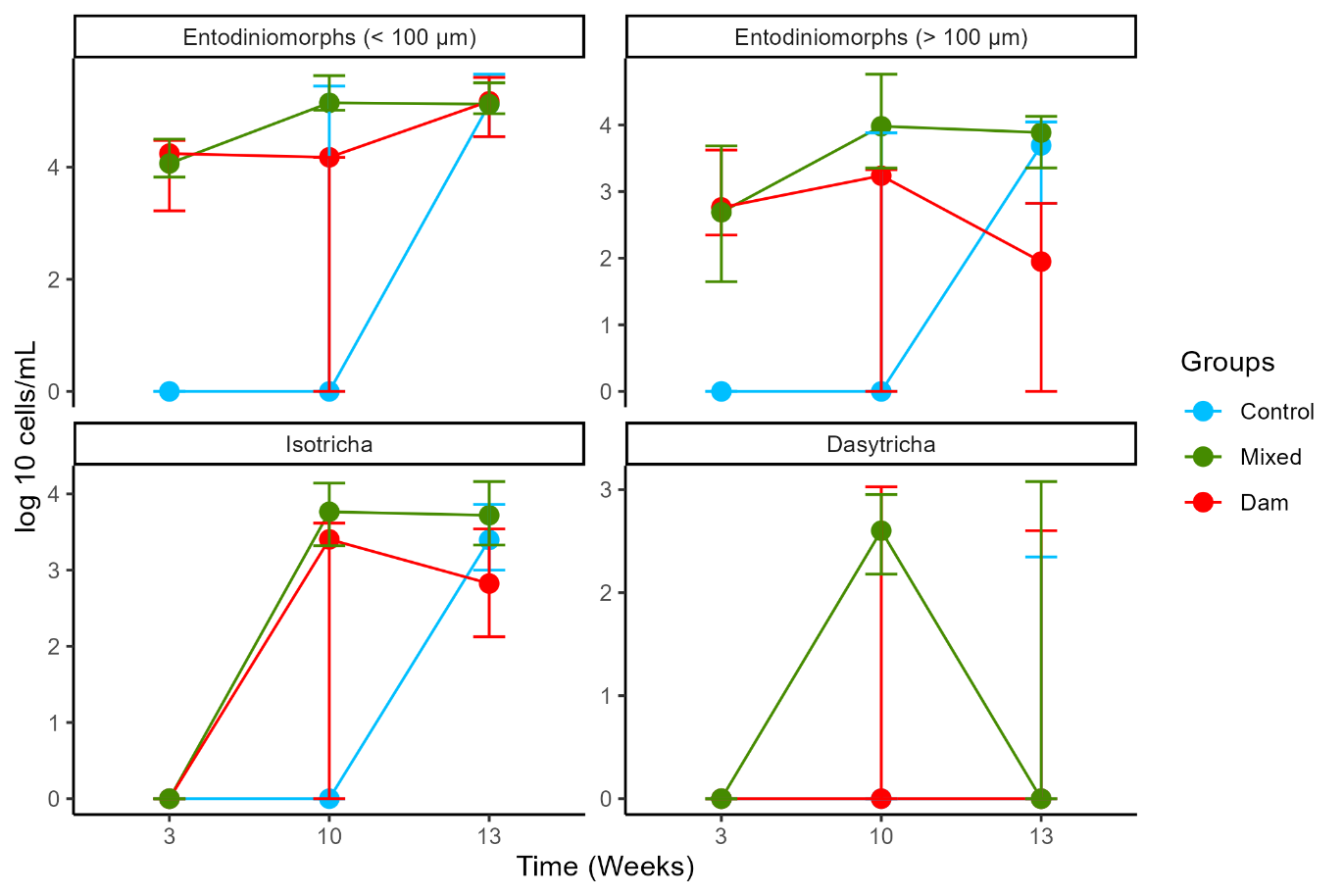


**Supplementary Figure 2**. Venn diagrams for each sampling week, showing the number and percentage of common and unique ASVs in the rumen communities of calves separated from dams at birth (Control, n= 9 for 3, 10 and 13 weeks and, n= 4 for 20 weeks), at the age of 4 weeks (Mixed, n= 7 for 3 weeks, n=9 for 10 and 13 weeks and, n= 8 for 20 weeks) or at weaning (Dam, n= 9 for 3, 10 and13 weeks and, n= 8 for 20 weeks)


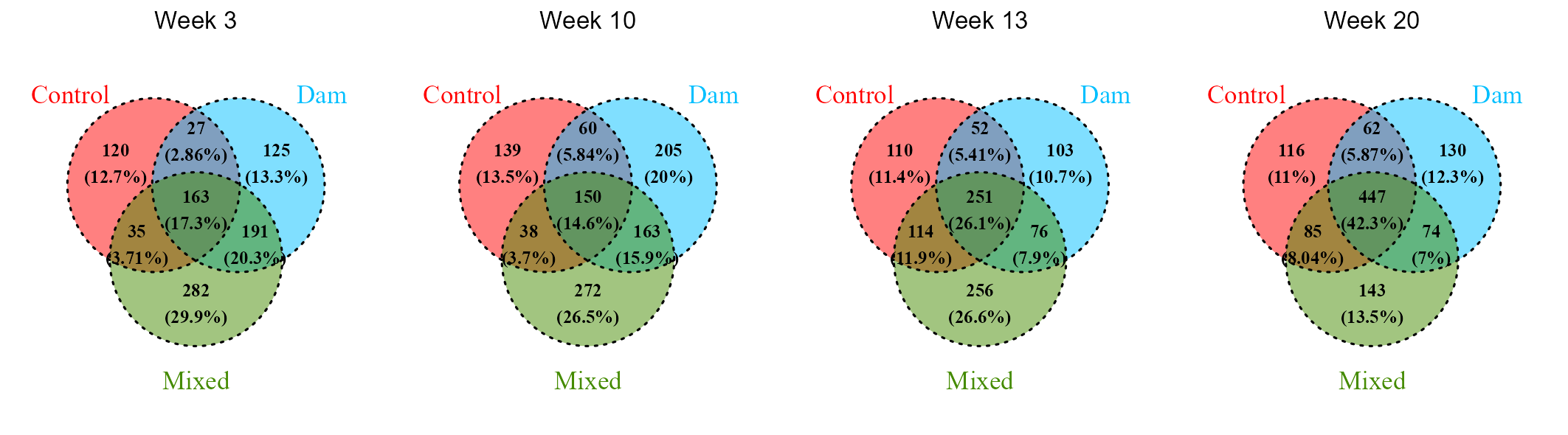


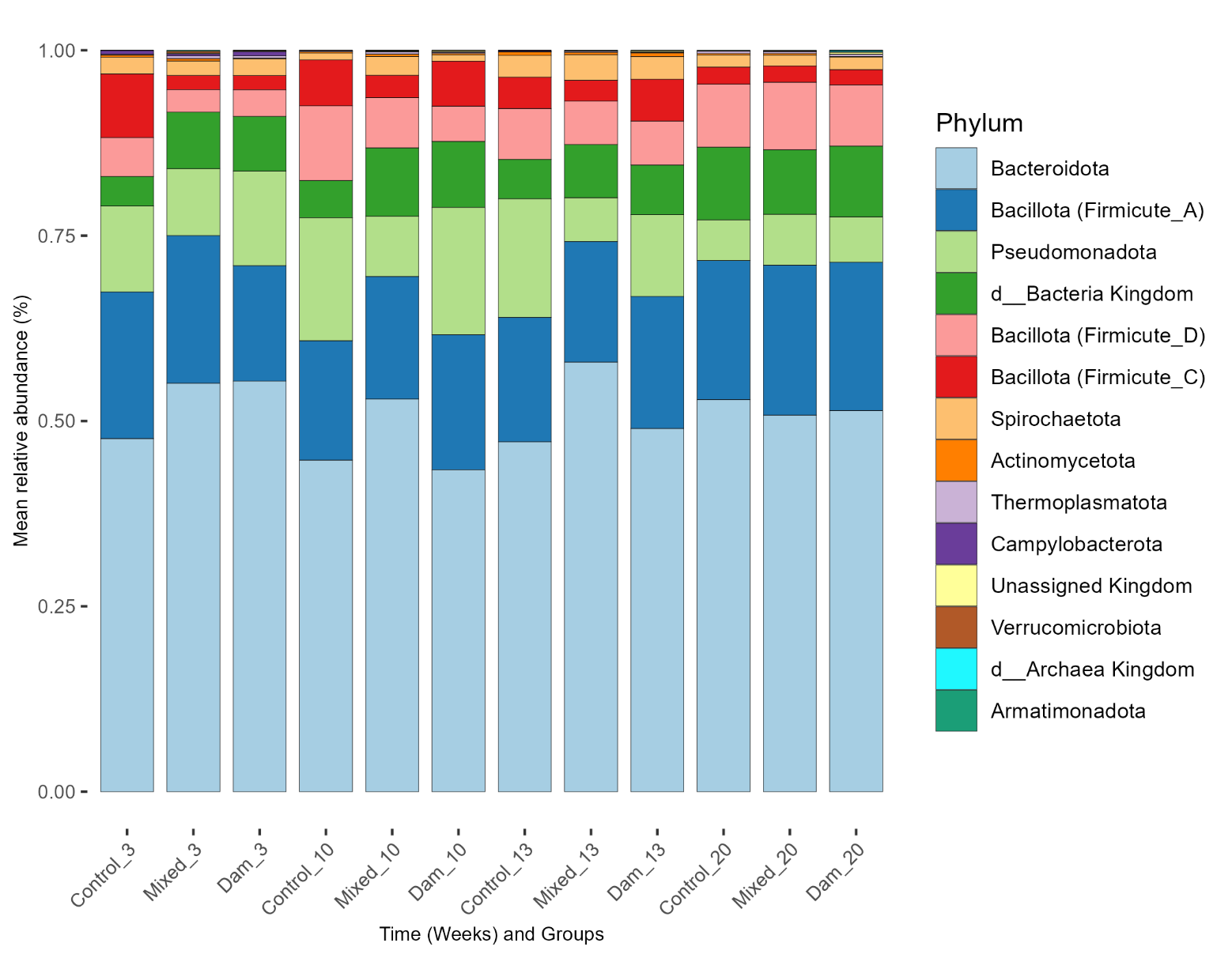
Supplementary Figure 3. Relative abundance of bacterial phyla (A) and genera (B), detected in the rumen communities of calves separated from dams at birth (Control, n= 9 for 3,10 and 13 weeks and, n= 4 for 20 weeks), at the age of 4 weeks (Mixed, n= 7 for 3 weeks, n=9 for 10 and 13 weeks and, n= 8 for 20 weeks) or at weaning (Dam, n= 9 for 3,10 and13 weeks and, n= 8 for 20 weeks). Values represent the mean for each group**.**

**(A)**


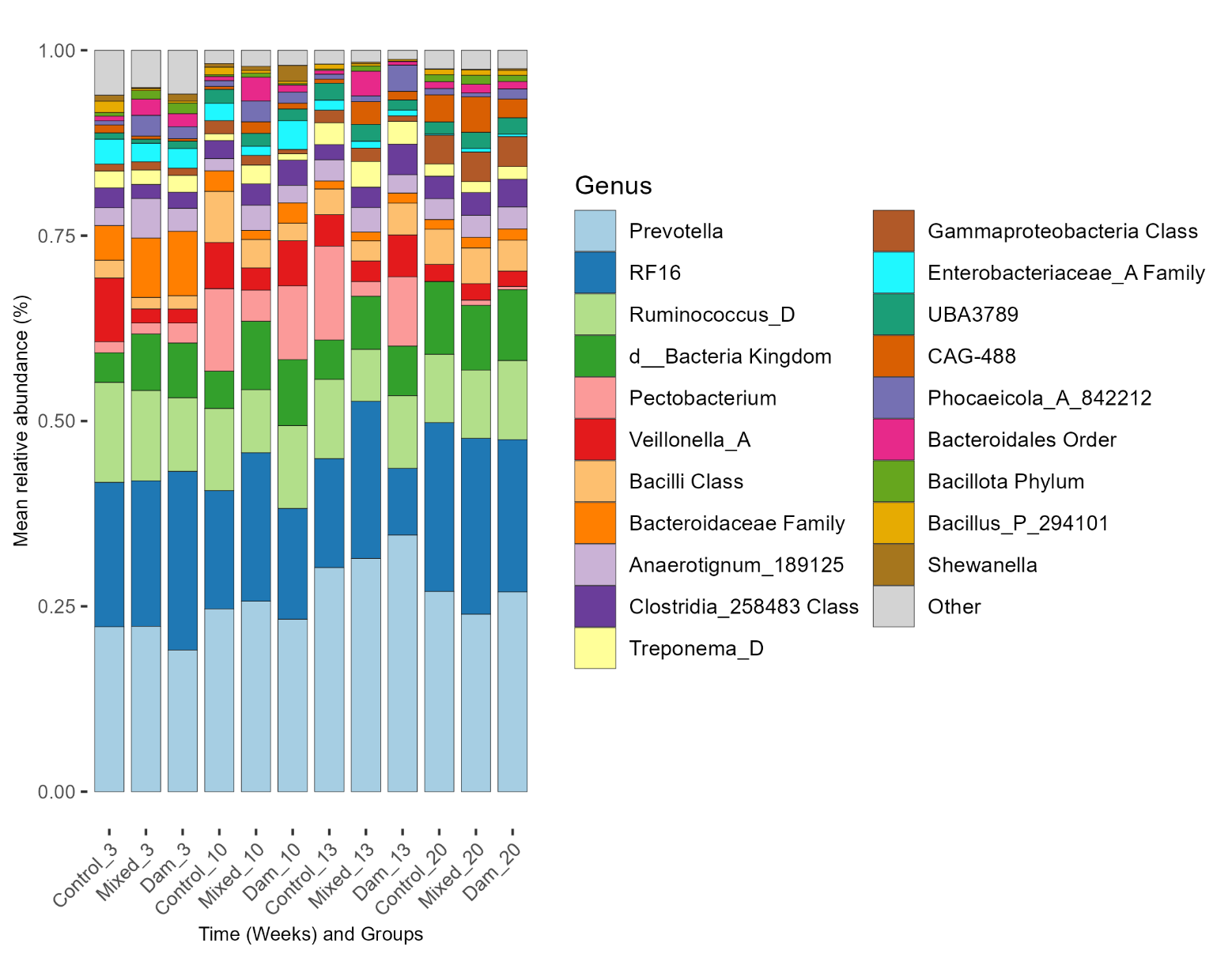


**(B)**

For more information on Supplementary Material and for details on the different file types accepted, please see [here](https://www.frontiersin.org/guidelines/author-guidelines#supplementary-material).
